# Information Source Characteristics of Personal Data Leakage During the COVID-19 Pandemic in China: Observational Study

**DOI:** 10.2196/51219

**Published:** 2024-12-10

**Authors:** Zhong Wang, Fangru Hu, Jie Su, Yuyao Lin

**Affiliations:** 1 School of Economics, Guangdong University of Technology, Guangzhou, China; 2 Key Laboratory of Digital Economy and Data Governance, Guangdong University of Technology, 161 Yinglong Road, Tianhe district, Guangdong province, Guangzhou, 510520, China, 86 18845127665; 3 School of Marxism, Peking University, Beijing, China

**Keywords:** public health emergency, privacy leakage, characteristics of information sources, COVID-19, China, information source, data privacy, public health, leakage

## Abstract

**Background:**

During the COVID-19 pandemic, in the period of preventing and controlling the spread of the virus, a large amount of personal data was collected in China, and privacy leakage incidents occurred.

**Objective:**

We aimed to examine the information source characteristics of personal data leakage during the COVID-19 pandemic in China.

**Methods:**

We extracted information source characteristics of 40 personal data leakage cases using open coding and analyzed the data with 1D and 2D matrices.

**Results:**

In terms of organizational characteristics, data leakage cases mainly occurred in government agencies below the prefecture level, while few occurred in the medical system or in high-level government organizations. The majority of leakers were regular employees or junior staff members rather than temporary workers or senior managers. Family WeChat groups were the primary route for disclosure; the forwarding of documents was the main method of divulgence, while taking screenshots and pictures made up a comparatively smaller portion.

**Conclusions:**

We propose the following suggestions: restricting the authority of nonmedical institutions and low-level government agencies to collect data, strengthening training for low-level employees on privacy protection, and restricting the flow of data on social media through technical measures.

## Introduction

Since December 2019, as COVID-19 cases increased worldwide, governments adopted numerous public health surveillance measures. Digital contact tracing using smartphone apps was endorsed by many governments, private firms, and universities as a potential tool to limit the spread of COVID-19 [1], but this raised privacy concerns since it can easily become a surveillance system if not properly designed and implemented [2]. However, in a public health emergency, a significant number of privacy issues are ignored under the pretext of efficiency and transparency [3]. In China, during this period, personal data leakage occurred frequently, and a large number of files containing personal data were widely shared on social media, resulting in widespread concerns. The perception of personal data protection has also been reshaped by COVID-19 [4]. The existing literature on personal data focuses more on the hazards of leaks and does not pay much attention to the information source.

The large-scale collection and use of personal data in public health emergencies begins with contact tracing technologies. Although contact tracing is a well-established part of the response to contagious disease outbreaks [5], it raises more privacy concerns when it is combined with big data technology. Big data technology has played an important role in the prevention and control of the COVID-19 pandemic in China, in the personal tracing, surveillance, and early warning of infectious diseases and the tracing of the virus’ sources as well as in drug screening, medical treatment, and resource allocation [6]. Personal data should undoubtedly be acquired, stored, and utilized for the effective prevention of pandemic situations. Information is the personal property of the user, but it is hosted on the server [7]. Even if personal data are incomplete, due to the iteration of big data technology, it can also bring huge economic benefits. Therefore, most motives behind privacy violations are for commercial purposes [8], including companies’ monitoring of personal data (shopping habits, social networking, etc), insiders’ misuse of personal data, the deliberate illegal sale of consumer data, and so on [9]. The reasons for data privacy breaches are classified into 4 categories, namely: technical failure (cybersecurity attacks), managerial failure (inefficient decision-making), organizational failure (incomplete and inconsistent policies), and human failure (loss or theft of documents or equipment) [9]. To account for the potential of malicious privilege abuse, the 2024 Data Breach Investigation Report released by Verizon adjusted their human factor calculations, yielding a more accurate estimate of the potential influence of security knowledge. Human causes were responsible for 68% of the breaches in the 2024 dataset [10]. Furthermore, with the development of research, the poor awareness of personal data protection has become recognized as one of the important reasons leading to privacy leakage [8].

Regarding the issue of privacy leakage, the existing literature mainly uses event research, content analyses, empirical analyses, and systematic reviews to study the harm caused by personal data leakage to listed companies [9,11], product users [12,13], and the general public [14]. Meanwhile, researchers have proposed corresponding countermeasures and suggestions, including strengthening technology [15], improving laws and regulations [7], formulating information protection standards and recovery mechanisms after the leakage, strengthening personal privacy concepts and training the skills of personal privacy [16], and so on. Specifically for health care organizations, Ignatovski [17] examined the differences in breach type and location in for-profit and nonprofit health care organizations and found that data breaches in for-profit organizations were more often due to theft, while they were commonly due to unauthorized access in nonprofit organizations.

The existing literature on privacy protection in public health emergencies mainly includes the following aspects of research: comparative analyses of privacy protection policies in public health emergencies in various countries or regions [4,18,19], research into privacy protection technology in public health emergencies [20], and applications and evaluations of health technology and informatics in public health emergencies [21]. Most of these study the impact of the incident or discuss how to manage it. However, there is a lack of research about the information source characteristics of privacy leaks in public health emergencies. Information necessitates an information source to ensure shareability owing to its abstract nature. Various types of documents and individuals in diverse positions are typically scrutinized as information sources [22]. In the context of personal data leakage, the information source pertains to the individual and the medium through which personal data were initially disseminated. The majority of existing literature pertaining to information sources predominantly emanates from the fields of informatics [22] and business, such as equity crowdfunding [23] and entrepreneurial performance [24]. However, there is a noticeable scarcity of research on public health information sources. Zhao et al [25] have made contributions to the literature by categorizing public health information sources into government, community, and personal domains. Their research delves into the examination of how usage patterns of information sources impact the self-protective behaviors of individuals endorsing COVID-19 misinformation. Exploring the characteristics of the information source in personal data leakage aids in pinpointing vulnerabilities in data security and effectively mitigating the risk of data breaches at the source. Given the rapid dissemination and expansive reach of information, addressing an information leakage becomes challenging without a comprehensive understanding of the information source characteristics. Drawing from existing research, our examination will encompass factors such as the organizational type and level of the leaker, the position held by the leaker, and the methods employed for data leakage during the prevention and control of the COVID-19 pandemic.

## Methods

### Ethical Considerations

The paper in question is excluded from ethical review since it does not include human subjects and the data used do not contain sensitive personal information or commercial interests, as per the Measures on Ethical Review of Life Science and Medical Research Involving Humans.

### Data Collection

From October 29 to 31, 2022, the original data were collected through Baidu’s advanced search engine, with the search terms “information,” “data,” “leakage,” “pandemic,” and “pandemic prevention,” and the search time was “all time.” Since there was a significant amount of duplicate and invalid information in these search results, the 2 researchers browsed the top 200 pages and saved the cases that were initially judged to be valid.

The sample selection followed the following principles: (1) information must come from official websites and (2) samples should contain information on at least 2 examination factors, such as the leaker’s job position, work organization, or leakage technique. Samples that met the above principles were included in the study.

It should be emphasized that all official announcements we collected were rigorously reviewed and cross-checked to verify their legitimacy and relevancy. When reviewing and interpreting the official announcements collected, we took extra effort to ensure that the title, language, and any claims were correct so that they would not mislead readers or create ambiguity about the research findings. Despite our best attempts to collect as many official notifications as possible through a thorough online search, some may still have been missed, particularly those published on nonmainstream websites or platforms. Furthermore, the choice of search keywords may have influenced the comprehensiveness of search results. As a result, these potential limitations should be considered when interpreting the findings of this study. Following thorough discussion, the 2 researchers retained and meticulously analyzed 40 cases, as detailed in Multimedia Appendix 1.

### Analysis

The source of the information leakage is the information controller, so the source characteristics include not only the demographic characteristics of the controller but also the social characteristics of their organization. Due to the different disclosure characteristics of each case, the main categories were summarized through open coding to extract the greatest extent of feature information. The coding work of this paper was completed with the help of ATLAS.ti software. The case text was imported into the analysis software. One researcher conducted an open coding analysis of the text, and the other researcher then reviewed it. If there were different opinions, they were unified after negotiation. After open coding, the researchers analyzed and organized the codes, and the main categories summarized are shown in Table 1. The data period spans from 2019 to 2022. Due to insufficient information in the cases, the demographic characteristics were omitted.

**Table 1.** Main category coding and the corresponding concept.

Number	Main category	Concept
G1	Type of organization	The type of organization where the leaker works, including government agencies, medical institutions, communities (village committees), companies, or schools.
G2	Administrative level of organization	The administrative level of the organization where the personal data breach occurred, such as the prefecture (eg, regions, cities, leagues), county (eg, districts, county-level cities), or township (eg, towns, streets).
G3	Post or rank	The rank of the leaker, such as junior staff member, mid-level employee, or person in charge.
G4	Employment type	The employment type of the person involved, such as supernumerary staff member, regular employee, or volunteer or temporary worker.
G5	Method of divulgence	How the data spread, such as through mobile phone photographs, screenshots, or forwarded files.
G6	Platform of divulgence	Where the data spread, such as in a friends’ group chat on WeChat, in a QQ group, or on a microblog.

## Results

After classifying the codes into the major categories, the coded data were subjected to a 1D matrix analysis (Tables2  3) as well as a 2D matrix analysis (Tables4  5), which revealed the following information source characteristics of personal data leakage.

**Table 2.** Coding and statistical analysis of organizations.

Main category and code		Organizations, n (%)
Type of organization (n=39)		
Government agency		23 (59)
Medical institution		6 (15)
Community		4 (10)
Company		3 (8)
School		3 (8)
Administrative level of organization (n=29)		
Township (town, street)		22 (76)
County		6 (21)
Prefecture		1 (3)

**Table 3.** Coding and statistical analysis of leakers^a^.

Main category and code		Leakers, n (%)
Post or rank (n=40)		
Junior staff member		26 (65)
Person in charge		10 (25)
Mid-level employee		4 (10)
Employment type (n=46)		
Regular employee		39 (85)
Volunteer or temporary worker		4 (9)
Supernumerary staff member		3 (7)

a The overall number of personal data breaches was 51 in all 40 cases, as several cases included more than one leaker. All of the leakers belonged to 39 different institutions. In some circumstances, coding values are absent owing to insufficient information.

**Table 4.** Coding and statistical analysis of divulgence method.

Main category and code		Methods or platforms, n (%)
Method of divulgence (n=43)		
Forwarded files		29 (67)
Photographs		11 (26)
Screenshots		3 (7)
Platform of divulgence (n=54)		
WeChat		52 (96)
QQ		1 (2)
Microblog		1 (2)

**Table 5.** Subdivision and statistical analysis of the WeChat platform.

Main category and code		Wechat Groups (n=52), n (%)
Other groups		18 (35)
Family groups		12 (23)
Work groups		7 (14)
Friend groups		4 (8)
Community groups		2 (4)
Private WeChat		8 (15)
Moments		1 (2)

### 1D Matrix Analysis

#### Organizational Characteristics of Privacy Leakage

As shown in Table 2, government agencies and medical institutions were the main sources of risk, and the lower the administrative level, the more likely someone in the organization was to leak personal data.

The government agencies with leaked data included health management departments, such as the District Health Bureau in C5 (Case 5; “C#” represents the cases involved in this study; please see Multimedia Appendix 1 for more details). The leaders of the health management departments, such as the C5 deputy director and the C4 deputy mayor, and the staff of subdistrict offices, such as that in C8, had access to the relevant personal information. Usually, the personal data of residents were directly stored in the staff member’s mobile phone and transmitted through WeChat. For example, a C10 community worker forwarded a “list of 35 close contacts” that he was strictly prohibited from forwarding in his WeChat work group chat.

There were relatively few cases of personal data leakage in organizations at high administrative levels. Leakage incidents mainly occurred in lower-level organizations but not in provincial organizations. Those at the county level included Wenshan Prefecture People’s Hospital in C3 and Qingdao Jiaozhou Central Hospital in C7, and those at the township level included C5 Yiyang Heshan District Health Bureau, C6 Yanjiao High-tech Zone Subdistrict Office, and C4 Hecheng Town Office. Organizations at higher administrative levels have a more standardized management system. Moreover, the higher the organization’s administrative level, the higher the overall quality of its employees. Thus, leakage incidents had a clear relationship with the professionalism and overall quality of the staff.

#### The Leaker

Employees at all levels can be leakers, and while the overall number of leakages by people in charge should be smaller, leaks occurred at a higher rate by people in junior staff and leadership posts than by mid-level employees.

The number of junior staff members is larger, and it is normal for more leaks to occur at this level. The disclosure of personal data by a person in charge or mid-level personnel also occurs from time to time but only accounts for about one-fifth of leakage cases. For example, in C5, the deputy director of Heshan District Health Bureau of Yiyang City forwarded the internal working documents to a staff member of the financial evaluation unit of the Finance Bureau through WeChat, who then spread it to WeChat groups.

Volunteers and temporary workers were also able to access summary documents containing a large amount of personal data. For example, as a volunteer, a street resident in C8 accessed the information of relevant personnel under investigation and sent it to WeChat groups, resulting in the large-scale dissemination of personal information. Three supernumerary employees in C3 publicly disseminated patient personal data.

#### Method of Divulgence

The main leakage platform was WeChat. The primary method was via file transfer; screenshots and images made up a very small percentage of leakage incidents.

More and more individuals are utilizing WeChat to send work-related data. For instance, in C15, a Hangzhou Hospital doctor forwarded the flow investigation report of an asymptomatic sick person on a WeChat group, and in C16, a firm employee secretly photographed and leaked an epidemiological investigation of a patient to a WeChat group.

Documents were the primary medium for data that were collected to prevent and control the spread of the COVID-19 pandemic. A large number of Excel or Word files containing epidemiological reports and patient information were disseminated on WeChat, and the ease of forwarding the files reduced the cost of leaking personal data. When a leaker does not have direct access to these documents, they may take photographs using their mobile device. For instance, during meetings, 3 officials from C13 utilized their smartphones to record internal information on the outbreak, and personnel from medical facilities in C3, C38, and C39 took photographs of patients’ medical records on hospital computers and leaked them. Even though the forwarding feature for files has limitations, the leaker can still reveal the data by taking screenshots. For instance, a staff member in C33 sent a work group chat screenshots of internal files related to a COVID-19 infection.

### 2D Matrix Analysis

#### Method of Divulgence and Type of Organization

The primary disclosure methods were the forwarding of documents in government agencies and mobile phone photography in medical institutions. Of the 24 occurrences involving information leaks from government agencies, 71% (n=17) involved document forwarding. Of the 5 leaks from medical institutions, 80% (n=4) used images taken on a phone (Table 6).

**Table 6.** Type of organization and method of divulging^a^.

Type of organization	Photographs, n (%)	Screenshots, n (%)	Forwarded files, n (%)
Government agency (n=24)	6 (25)	1 (4)	17 (71)
Medical institution (n=5)	4 (80)	0 (0)	1 (20)
Community (n=3)	0 (0)	0 (0)	3 (100)
Company (n=5)	1 (20)	3 (60)	1 (20)
School (n=2)	0 (0)	1 (50)	1 (50)

a Some cases lacked information about a certain dimension, so they were not counted.

#### Method of Divulgence and Administrative Level of Organization

The results showed that high-level organizations have a lower rate of personal data leakage through the forwarding of documents. There were 2 out of the 5 incidents in district institutions, accounting for 40% of information leakage incidents, and 16 out of the 24 cases in township institutions, accounting for 67% (Table 7). The fact that the leaker could directly forward files containing sensitive data indicates that the organizations lacked basic permission management. The level of data management at an organization was thus initially assessed by the percentage of forwarded documents.

**Table 7.** Administrative level of organization and method of divulging.

Administrative level of organization	Photographs, n (%)	Screenshots, n (%)	Forwarded files, n (%)
Prefecture (n=1)	1 (100)	0 (0)	0 (0)
County (n=5)	3 (60)	0 (0)	2 (40)
Township (town, street; n=24)	7 (29)	1 (4)	16 (67)

#### Method of Divulgence and Post or Rank

People in charge and mid-level personnel mainly leaked personal data by forwarding files or taking photographs; they rarely used screenshots. This is perhaps due to it being easier for higher-level personnel to bypass permission requests and directly access personal data files. The proportion of low-level employees leaking personal data by forwarding files was relatively low, though some used the screenshot function to cause a leakage (Table 8).

**Table 8.** Post or rank and method of divulging.

Post or rank	Photographs, n (%)	Screenshots, n (%)	Forwarded files, n (%)
Person in charge (n=9)	3 (33)	0 (0)	6 (67)
Mid-level employee (n=3)	1 (33)	0 (0)	2 (67)
Junior staff member (n=28)	6 (21)	6 (21)	16 (57)

## Discussion

### Laws and Regulations for Health Care Data Protection

In terms of health care data protection, China primarily relies on laws and regulations such as the Cybersecurity Law, the Data Security Law, the Personal Information Protection Law, and the Cybersecurity Management Measures for Medical and Health Facilities. These regulations establish specific security criteria for all stages of medical data collection, storage, processing, and transmission, as well as consequences for data breaches. For example, Article 253 of the Criminal Law clearly specifies criminal culpability for leaking citizens’ personal information (including medical data), and if the circumstances are serious, the culprit may be sentenced to fixed-term imprisonment of not more than 3 years or criminal detention. Overall, China is continuously improving its relevant laws and regulations to curb data leaks, and its experience holds referential value for other countries [26].

The Health Insurance Portability and Accountability Act (HIPAA) governs the majority of health care data protection in the United States. HIPAA establishes standards for health care information privacy and security, requiring health care organizations, health insurance companies, and other regulated entities to take adequate measures to secure individuals’ personal health information. HIPAA violations can be investigated and penalized by entities such as the Federal Trade Commission and the Department of Justice and can result in significant fines and criminal liability. The General Data Protection Regulation (GDPR) serves as the foundation for EU health care data privacy laws. The GDPR requires businesses and organizations to process personal data in a transparent, equitable, and lawful manner, setting high requirements for the protection of personal data within the European Union. Data protection authorities in EU member states possess the authority to look into and apply sanctions for GDPR violations, which can include steep fines of up to 4% of annual turnover or €20 million (US $21.12 million), whichever is larger [27].

### Interpretation and Policy Recommendations

#### Principal Findings

We investigated the information source characteristics of personal data leakage in a public health emergency and have the following findings:

1. Personal data leakage incidents in a public health emergency were more likely to occur in organizations at lower administrative levels or those with less regulated data management. In stark contrast, medical institutions, which possess the most comprehensive personal data, experienced a relatively low incidence of data breaches. The nearly universal recognition of medical oaths and codes of duty-based ethics to protect patient privacy and confidentiality is a powerful indicator of the moral significance of these concepts [28]. Provisions on Medical Records Management of Medical Institutions, Article 1226 of the Civil Code of the People’s Republic of China, stipulates that medical institutions and their medical personnel shall keep the privacy and personal data of patients confidential [29]. When medical staff obtain professional qualifications, they shall also have relevant training and education in protecting patients’ personal data.
2. The primary disclosure techniques were the forwarding of documents in government agencies and mobile phone photography in medical institutions. Medical institutions have implemented several steps to limit access to information, such as access limitations, access rights restrictions, access location restrictions, access content restrictions, and patient-informed consent guidelines [30]. Standardized permission management in health care organizations makes direct data leakage difficult, and most leakers can only disseminate personal data indirectly by taking pictures. The use of WeChat for business purposes, which is more widespread in China, and the lack of restrictions on internal file transfers and sharing have resulted in an increase in privacy leakage cases.
3. Lower-level employees were more prone to violate personal data due to a lack of discipline or privacy awareness. The main way that middle and senior management disclosed personal data was by forwarding it to unauthorized personnel and then disseminating it through them. Therefore, the ability to forward data should be restricted to middle- and high-level staff who should be allowed only to transfer files internally, not externally or to unauthorized personnel. The outbreak of the COVID-19 pandemic reduced manpower in many organizations, meaning that a large amount of personal data were often processed by supernumerary staff or even temporary workers. The staff responsible for investigating the pandemic were not adequately trained and lacked strict supervision over the collection, storage, and forwarding of personal data.
4. Online social networks (OSNs) have become an essential element in modern life for people to stay connected to each other [31]. Microblogging is a typical OSN service in China. Although WeChat and QQ belong to private instant messaging apps, which are not exactly the same as OSNs, their derivative functions such as channels and subscriptions have OSN attributes. Additionally, these apps have a high level of popularity and usage. When private, sensitive information is shared on these 2 sites, it has a significant negative impact. Various OSN platforms can easily become channels for spreading personal data; this is especially true for WeChat as well. The photograph and screenshot functions of mobile phones make it easier for staff members to become personal data leakers. Restricting a mobile phone’s photograph and screenshot functions in specific scenarios could reduce the occurrence of personal data leakage cases.

Some studies have shown that the main and most important factor to ensure the security of information systems is the human factor [32]. According to our results, the main causes of personal data leakage are analyzed from the information source perspective as follows. First, there is a lack of effective rights management. Many organizations or individuals have the right to control data, but their obligations and responsibilities are not clear, so the rights are abused. Second, the privacy awareness of those who control sensitive data is inadequate. Organizations or individuals who do not have the relevant skills and capabilities have the right to control data, which is more likely to lead to data dissemination in violation of regulations. Third, technical means are unsophisticated. In the process of data collection, transmission, and processing, there are many means used, such as manual forms, personal mobile phones, and personal WeChat channels. Even if there is no illegal disclosure, data can easily be stolen by hackers. The collector, maintainer, or processor of personal data (hereinafter referred to as a controller) may be the source of information disclosure. Drawing from this, the paper proposes the following recommendations.

#### Restricting the Data Collection Authority of Nonmedical Institutions and Low-Level Government Agencies

At present, many scholars have done relevant research on the collection of personal data and on the GDPR’s strict regulations of such collection [33-35]. The occurrence of these data leakage cases often comes from the unclear division of the rights and responsibilities of the personal data controller. For example, there is no clear regulation of the authority of certain community or company staff members to collect personal data or of the handling of such data. Therefore, it is particularly critical to clarify the rights and responsibilities of personal data controllers in public health events.

Big data analysis of disease prevention and control involves a large amount of personal data, including tracking analyses of specific populations, which are not authorized to be carried out by any unit or individual [36]. At present, streets, neighborhood committees, and medical institutions have collected a large amount of personal data, including the name, gender, age, address, ID number, mobile phone number, recent travel history, contact history, and health status of individuals and their family members. Excessive data collection not only increases the workload of collectors but also increases the cost of data security maintenance and the risk of leakage. One solution is reducing unnecessary data collection, such as reducing the amount of mobile phone or telephone numbers collected or prohibiting the collection of ID numbers. This would not only reduce workload and improve the cooperation of data subjects but hackers would also be less motivated to steal data. Furthermore, if the data are leaked, the losses would be greatly reduced.

#### Strengthening Training on Privacy Protection for Low-Level Employees

Individuals can be regarded as data controllers and should comply with relevant laws and regulations pertaining to social networks [37]. Many domestic laws and regulations exist in China, such as the Cybersecurity Law of the People’s Republic of China, the Law of the People’s Republic of China on the Prevention and Control of Infectious Disease, Regulation on Public Health Emergencies, and so on. They protect personal privacy, especially during public emergencies, by putting forward the relevant requirements, clarifying punishments for the disclosure of personal information in different scenarios, and providing legal support to protect the legitimate rights and interests of individuals. However, some government workers do not understand these laws and regulations or do not pay attention to them.

The first suggestion is to carry out special training on privacy protection. Some staff members sent documents or screenshots containing important information to WeChat work groups or to their relatives and friends. It is recommended that special training be carried out to urge all government staff to attach importance to personal privacy protection. The second recommendation is to establish a privacy review mechanism for important positions. Since a lack of privacy awareness was closely related to data leakage behaviors, it is important to determine and ensure an adequate level of awareness [38]. For positions that are required to deal with a large amount of personal data over a long period of time, we recommend regular privacy reviews to assess the implementation of personal data protection regulations and the level of awareness of the people involved.

#### Restricting the Flow of Data on Social Media Through Technical Means

From previous studies, the methods to protect personal data from a technical perspective include k-anonymity [39], l-diversity [40], t-closeness [41], differential privacy [42], identity-based anonymization [43], and other new technologies [44]. In the case of this study, the leaking of personal data through Excel and Word documents and mobile phone photographs reflects an outdated method of information processing in the current COVID-19 pandemic prevention work. The digitization process of pandemic prevention should be accelerated, and big data technology should be fully utilized to ensure safety while improving efficiency.

We advise against sharing confidential files on social media and, in some circumstances, we advise limiting the use of screenshots and images on employees’ phones. This can be done through the development of professional information systems, data entry, and centralized management of mobile phones or other personal communicators. In this way, data access, usage, and download conditions can be gained through permission requests and identity authentication, which can effectively curb the release of data by locking out the violator. The government can formulate personal data classification standards based on the sensitivity of the data or the degree of the security risk and strictly regulate highly sensitive data such as medical personal information [45]. Furthermore, a professional anti–privacy leakage team can be built for professional information systems, which will make it harder for hackers to steal information. Finally, a data breach announcement policy should be established. For instance, HIPAA in the United States stipulates that in the event of a data breach, the data subjects must be notified immediately, which can help mitigate the losses for these individuals.

### Strengths and Limitations

Personal data protection is an important part of the public health emergency management system, but the relevant research is inadequate. To the best of our knowledge, this is the first paper to investigate the information source characteristics of personal data leakage during the COVID-19 pandemic, which has crucial implications for future privacy protection during public health emergencies. In addition, the research method of text analysis of real data leakage cases adopted in this paper is innovative and can provide ideas for subsequent related research.

Several limitations of this study need to be mentioned. First, the lack of sufficient samples may cause a bias in the statistical results and affect the reliability of conclusions. Second, media coverage of personal data breach cases may have been selective, and some cases involving sensitive topics may have been hidden. This would have reduced the randomness of the cases collected in this paper. Further studies could analyze more cases to describe the information source characteristics of personal data leakage more accurately in public health emergencies.

### Conclusion

The results showed that personal data leakage in a public health emergency occurred more in organizations at lower administrative levels or those with less regulated data management. Perhaps due to a lack of discipline or privacy awareness, there were more personal data breaches associated with lower-level employees. Therefore, standardizing data management in lower-level organizations and raising the privacy awareness of junior staff members are crucial to protecting personal data. Various OSN and instant messaging platforms, especially WeChat, can easily become channels for diffusing personal data. The photograph and screenshot functions of mobile phones made it easier for staff members to become personal data leakers. Restricting the phone’s photograph and screenshot functions in specific scenarios may reduce the occurrence of personal data leakage cases. These findings fill a gap in the literature, demonstrating what type of organization or individual is the weak point for personal data protection and how personal data are leaked during a public health emergency.

## Supplementary material

10.2196/51219 Multimedia Appendix 1 Research data.

## Abbreviations

GDPR: General Data Protection Regulation
HIPAA: Health Insurance Portability and Accountability Act
OSN: online social network
