# Supplementary material for: Information Source Characteristics of Personal Data Leakage During the COVID-19 Pandemic in China: Observational Study
Source: JMIR Med Inform. 2024 Dec 10;12:e51219. doi: 10.2196/51219 (PMC11651669; doi:10.2196/51219)
Supplement: Multimedia Appendix 1 [file medinform-v12-e51219-s001.docx]

# Appendix

Appendix A Cases of Personal Data Breach Involved in the Epidemic, China, 2019-2022

| **Number** | **Name** | **Abstract** | **Source(latest visit date from October 29 to October 31, 2022）** |
| --- | --- | --- | --- |
| C1 | Personal data leakage in Tianzhu County, Guizhou Province | Divulged the list of relevant personnel and their personal data in a certain street involved in the prevention and control of the epidemic situation | <https://www.sohu.com/a/371775402_674455> |
| C2 | Personal data leakage in Zhoushan City, Zhejiang Province | Divulged a piece of data about people of Hubei nationality | <https://www.sohu.com/a/372414508_120214179> |
| C3 | Personal data leakage at Wenshan People’s Hospital | Divulged the name, home address, work unit, itinerary, contact person, diagnosis, and treatment data of the confirmed case | <http://www.yn.xinhuanet.com/newscenter/2020-02/08/c_138765428.htm> |
| C4 | Personal data leakage in Zixi County, Jiangxi Province | Divulged the personal data of Chen, a confirmed case of pneumonia, and 29 people in close contact with him | <https://www.sohu.com/a/370174253_120207595> |
| C5 | Personal data leakage in Yiyang City, Hunan Province | Divulged the personal privacy data of 11 people, including Zhang and his relatives, in the investigation report of pneumonia cases | https://baijiahao.baidu.com/s?id=1734378315739534917&wfr=spider&for=pc |
| C6 | Personal data leakage in a community in Guangming District of Shenzhen | Divulged community residents' names, ID numbers, addresses， telephone numbers, etc. | <http://www.sznews.com/news/content/2020-02/16/content_22868735.htm> |
| C7 | Personal data leakage in Qingdao, Shandong Province | Divulged personal identity data such as names, addresses, contact data, and ID card numbers of more than 6000 people | <http://www.xinhuanet.com/2020-04/19/c_1125877355.htm> |
| C8 | Personal data leakage in Yanjiao City, Hebei province | Divulged the personal data, whereabouts, and health status of the three members of Zhang's family | <https://www.sohu.com/a/403068172_161795?_trans_=000014_sgss_sgnbaxw> |
| C9 | Personal data leakage in Ordos City, Inner Mongolia | Divulged the personal data of the personnel involved in the investigation of the epidemic situation | <http://www.nmg.xinhuanet.com/xwzx/2020-02/06/c_1125535156.htm> |
| C10 | Personal data leakage in Linfen City, Shanxi Province | Divulged the personal data of 35 close contacts, such as their names, identity numbers, home addresses, and so on | <https://www.sohu.com/a/370180920_255783> |
| C11 | Personal data leakage in Ningbo City, Zhejiang Province | Divulged the personal data of the confirmed patient Sun and his relatives | <https://www.sohu.com/a/375306309_260616> |
| C12 | Personal data leakage in Liangshan Prefecture, Sichuan Province | Divulged the personal data of COVID-19's epidemic prevention personnel | <https://www.sohu.com/a/371846729_116237> |
| C13 | Personal data leakage in Chengdu Epidemic Prevention and Control Work Scheduling Meeting | Divulged the epidemic prevention and control work data | <https://www.163.com/dy/article/HHPEMQA30511BI8B.html> |
| C14 | Personal data leakage in Yibin City, Changning Province | Divulged the epidemic prevention and control work data | <https://m.thepaper.cn/baijiahao_20007083> |
| C15 | Personal data leakage at a hospital in Hangzhou | Divulged the personal data of asymptomatic infected patients in an epidemiological investigation | <https://mp.weixin.qq.com/s?__biz=MjM5NDkzNDU4Ng==&mid=2649982862&idx=8&sn=1f9cca7f74998734a8accf10bf544ae1&chksm=be871ddb89f094cd67713128006eaf4913e07a55722eccc47e38e95a25082116f102e07ab21e&scene=27> |
| C16 | Personal data leakage at an aviation security company in Beijing | Divulged the epidemiological investigation of patient | <https://mp.weixin.qq.com/s?__biz=MjM5NDkzNDU4Ng==&mid=2649982862&idx=8&sn=1f9cca7f74998734a8accf10bf544ae1&chksm=be871ddb89f094cd67713128006eaf4913e07a55722eccc47e38e95a25082116f102e07ab21e&scene=27> |
| C17 | Personal data leakage at CDC of a county in Shanxi | Divulged the epidemiological investigation of patient | <https://mp.weixin.qq.com/s?__biz=MjM5NDkzNDU4Ng==&mid=2649982862&idx=8&sn=1f9cca7f74998734a8accf10bf544ae1&chksm=be871ddb89f094cd67713128006eaf4913e07a55722eccc47e38e95a25082116f102e07ab21e&scene=27> |
| C18 | Personal data leakage in Dashiqiao City | Divulged the electronic files of epidemic prevention data (including personal ID numbers of citizens, home addresses, and other data) | <https://baijiahao.baidu.com/s?id=1732532984510333719&wfr=spider&for=pc> |
| C19 | Personal data leakage in Jishou City, Hunan Province | Divulged the epidemic prevention and control delivery list of Jishou City | <https://www.thepaper.cn/newsDetail_forward_20266780> |
| C20 | Personal data leakage at a certain CDC of Nandan county in Guangxi | Divulged the work materials related to the prevention and control of the new coronavirus | <https://www.thepaper.cn/newsDetail_forward_20266780> |
| C21 | Personal data leakage at a certain CDC of Liujiang district in Guangxi | Divulged the content involving confidential investigation reports | <https://www.thepaper.cn/newsDetail_forward_20266780> |
| C22 | Personal data leakage at Fuchuan County People’s Hospital in Guangxi | Divulged the privacy of persons involved in the epidemic (patient medical records) | <https://www.thepaper.cn/newsDetail_forward_20266780> |
| C23 | Personal data leakage in Zhongshan County, Guangxi Province | Divulged the documents containing data on people returning to or passing through Guangxi from Hubei | <https://www.thepaper.cn/newsDetail_forward_20266780> |
| C24 | Personal data leakage in Wuda District of Nei Monggol | Divulged personal data of close contacts | <https://www.thepaper.cn/newsDetail_forward_20266780> |
| C25 | Personal data leakage in Hailin City | Divulged personal data such as the names, identity card numbers, telephone numbers, and home addresses of 9 epidemic-related close contacts | <https://baijiahao.baidu.com/s?id=1730438703483824899&wfr=spider&for=pc> |
| C26 | Personal data Divulgeage incident in Shenyang City | Divulged personal data of close contacts | <https://baijiahao.baidu.com/s?id=1728100245577138140&wfr=spider&for=pc> |
| C27 | Personal data Leakage in Guilin City | Divulged sensitive data of epidemic personnel | <http://k.sina.com.cn/article_5943880432_162486af0001011atl.html> |
| C28 | Personal data Leakage in Lianyuan City | Divulged the personal data of new coronavirus infections | https://cj.sina.com.cn/articles/view/2675303511/9f75e45701900zbaf |
| C29 | Personal data Leakage in Gushi County | Divulged on-site epidemiological survey personnel initially formed work data that was not confirmed and officially released by authoritative departments | https://view.inews.qq.com/a/20220506A05TWN00 |
| C30 | Personal data Leakage at a certain unit in Hangzhou City | Divulged the nucleic acid test results of the tested person | https://baijiahao.baidu.com/s?id=1715391762518285550&wfr=spider&for=pc |
| C31 | Personal data Leakage in Lianyungang City | Divulged three internal documents on epidemic prevention and control work | https://baijiahao.baidu.com/s?id=1727090284705637182&wfr=spider&for=pc |
| C32 | Personal data Leakage of Donghai County Transportation Bureau | Divulged the internal documents involved in the epidemic | https://baijiahao.baidu.com/s?id=1727090284705637182&wfr=spider&for=pc |
| C33 | Personal data Leakage of Donghai County Telecom Co. | Divulged the internal documents involved in the epidemic | https://baijiahao.baidu.com/s?id=1727090284705637182&wfr=spider&for=pc |
| C34 | Personal data Leakage in Tang County of Baodin City | Divulged close contacts initial nuclear data table ( including personnel household address, name, identity card number, telephone number ) | https://baijiahao.baidu.com/s?id=1689205776253453179&wfr=spider&for=pc |
| C35 | Personal data Leakage in Yanshi City, Henan Province | Divulged data about epidemic prevention and control-related personnel | https://baijiahao.baidu.com/s?id=1688579759875400677&wfr=spider&for=pc |
| C36 | Personal data Leakage in Xibuhe Country of Linlang County | Divulged Liang and others’ personal data | https://baijiahao.baidu.com/s?id=1688579759875400677&wfr=spider&for=pc |
| C37 | Personal data Leakage in Yiyang City, Hunan Province | Divulged epidemic data | http://www.hunan.gov.cn/hnszf/hnyw/szdt/202108/t20210803_20165062.html |
| C38 | Personal data Leakage of Pinghu Health and Wellness Bureau | Divulgeded medical records of confirmed cases of novel coronavirus pneumonia | https://baijiahao.baidu.com/s?id=1676097809341785440&wfr=spider&for=pc |
| C39 | Personal data Leakage in Lingao Towen of Baishui County, Shanxi Province | Divulged patient medical records | https://baijiahao.baidu.com/s?id=1689157627712667658&wfr=spider&for=pc |
| C40 | Personal data Leakage in Putian City | Divulged the personal data of the positive personnel and their close contacts in the preliminary screening of novel coronavirus nucleic acid test results | https://baijiahao.baidu.com/s?id=1711656975985522772&wfr=spider&for=pc |
